# Metabolomic Approaches in Cancer Epidemiology

**DOI:** 10.3390/diseases3030167

**Published:** 2015-08-11

**Authors:** Mukesh Verma, Hirendra Nath Banerjee

**Affiliations:** 1 Methods and Technologies Branch, Epidemiology and Genomics Research Program, Division of Cancer Control and Population Sciences, National Cancer Institute, National Institutes of Health, Suite 4E102, 9609 Medical Center Drive, Bethesda, MD 20892, USA; E-Mail: vermam@mail.nih.gov; 2 Department of Biological and Pharmaceutical Sciences, Elizabeth City State University, Campus Box 930, 1704 Weeksville Road, Elizabeth City, NC 27909, USA

**Keywords:** biomarker, cohort, epidemiology, metabolomics

## Abstract

Metabolomics is the study of low molecular weight molecules or metabolites produced within cells and biological systems. It involves technologies such as mass spectrometry (MS) and nuclear magnetic resonance spectroscopy (NMR) that can measure hundreds of thousands of unique chemical entities (UCEs). The metabolome provides one of the most accurate reflections of cellular activity at the functional level and can be leveraged to discern mechanistic information during normal and disease states. The advantages of metabolomics over other “omics” include its high sensitivity and ability to enable the analysis of relatively few metabolites compared with the number of genes and messenger RNAs (mRNAs). In clinical samples, metabolites are more stable than proteins or RNA. In fact, metabolomic profiling in basic, epidemiologic, clinical, and translational studies has revealed potential new biomarkers of disease and therapeutic outcome and has led to a novel mechanistic understanding of pathogenesis. These potential biomarkers include novel metabolites associated with cancer initiation, regression, and recurrence. Unlike genomics or even proteomics, however, the degree of metabolite complexity and heterogeneity within biological systems presents unique challenges that require specialized skills and resources to overcome. This article discusses epidemiologic studies of altered metabolite profiles in several cancers as well as challenges in the field and potential approaches to overcoming them.

## 1. Background

Metabolomics, the study of metabolites produced in the body, has the potential to be useful in identifying novel diagnostic biomarkers and understanding cancer etiology. Metabolomics is considered most closely related to a patient’s phenotype [1,2]. The metabolome is the complete set of small-molecule metabolites that are found in a biological sample, and the human metabolome contains approximately 2500 metabolites [3]. Like the transcriptome, epigenome, and proteome, the metabolome is dynamic and changes over time. Metabolomics is the quantitative assessment of endogenous metabolites within a biologic system. Knowing a metabolome may allow early cancer detection and diagnosis or it may be a predictive and pharmacodynamic marker of drug effects. Metabolic and molecular imaging technologies, such as positron emission tomography (PET) and MRI, enable noninvasive metabolic marker detection. The HIF-1 pathway affects response to radiotherapy by HIF-1 protection of vasculature after irradiation and the regulation of glycolysis and the pentose phosphate pathway, thus increasing tumor antioxidant capacity. [18 F]-fluorodeoxyglucose-PET images can be used to quantitatively determine glucose metabolic rate and pharmacokinetic rate constants in tissue volumes, which is useful for the radiotherapy pharmacokinetic analysis conducted to determine the rate constants of the fluorodeoxyglucose metabolism in 41 patients (104 lesions). The highest glucose metabolic rate tumor regions had high cellular uptake and phosphorylation rate constants with relatively low blood volume. In regions with less metabolic activity, the blood volume fraction was higher and cellular uptake and phosphorylation rate constants were lower. Thus, the tumor glucose phosphorylation rate was not dependent on nutrient transport [4,5].

Metabolomics may prove useful in following the effects of pathophysiological stimuli, as metabolomic profiling reflects changes that occur during disease development, progression, and response to therapy. Metabolomic biomarkers have been used in cancer surveillance [4]. Technologies such as mass spectrometry (MS) and nuclear magnetic resonance spectroscopy (NMR) can be used to analyze samples that are isolated from the biofluids and tissues of cancer cases and controls. Compared to genetic and proteomic approaches, metabolomics generates more complex data. Koo *et al.* proposed the construction of metabolomic association networks that use high-dimensional MS data [5,6]. Tissue-specific metabolites have been identified in different cancer samples [1]. It is important to note that metabolomic markers themselves are not considered to be tumor-specific, but their altered correlations (representing different pathways) lead to concentrations and patterns of metabolomic intermediates and end products that are specific to either patients or healthy individuals. 

Both targeted and untargeted (or non-targeted) approaches are applied in metabolomics and epidemiology. The untargeted approach applies when no prior knowledge is available about the biomarkers to be identified; the methods applied are used to detect all possible metabolites. The targeted approach applies when a small number of suspected metabolites need to be quantified so that cases and controls can be distinguished. When using metabolomics, large sample sizes are needed for successful epidemiologic studies with minimum variability [7]. Cohort consortia with thousands of samples are an excellent resource for such studies. Furthermore, metabolomics is a high-throughput technique that is cost-efficient, fast, and adaptable for epidemiologic studies.

## 2. Application of Metabolomic and Epidemiologic Studies to Selected Tumor Types

This section describes examples of selected tumor types for which metabolomic and epidemiologic studies have been conducted. The information provided could be useful in stratifying patients for a variety of tumor types based on their metabolomic profiling.

### 2.1. Bladder Cancer

Bladder cancer is the fifth most common cancer in the world and has a high rate of recurrence. The surveillance protocol for this cancer involves urine cytology and cystoscopy every three months for two years, every six months for the next two years, and then yearly thereafter [8,9]. Promising markers for bladder cancer include bladder tumor antigen, nuclear matrix proteins, minichromosome maintenance protein, cystatin B, profiling B, and a number of glycosylated proteins. In one epidemiologic study, bladder cancer cases and controls could be distinguished based on liquid chromatography (LC)-MS metabolomic profiling [10]. In this study, carnitine transferase and pyruvate dehydrogenase levels were much higher in cases compared to controls. Zhang *et al.* reported on the use of urinary modified nucleosides as biomarkers for monitoring urothelial bladder cancer [11].

### 2.2. Breast Cancer

Breast cancer accounts for the largest number of newly diagnosed cases in female cancer patients, and biomarkers are needed that can detect this cancer early and identify responders among patients undergoing treatment. In the United States, the number of breast cancer cases is projected to increase each year, which poses a burden for both the health care system and the economy. Tang *et al.* identified metabolites associated with breast cancer heterogeneity [12]. Gas chromatography (GC)-MS and LC-MS analysis of tumor samples characterized under The Cancer Genome Atlas (TCGA) project of the National Cancer Institute (NCI), National Institutes of Health (NIH), revealed that 18% of metabolites were present at higher levels in ER− breast cancer samples compared to ER+ samples. The main metabolites were glycolytic and glycogenolytic intermediates. Higher levels of glutathione in ER− breast cancer suggested the ability to handle oxidative stress and toxins better than ER+ breast cancer. In another study, the level of patient frailty was determined based on metabolomic profiling [13]. Some of the metabolites found to be altered in frail patients were amino acids, including serine, tryptophan, hydroxyproline, histidine, and its derivative 3-methylhistidine, cystine, and beta aminoisobutyric acid. A prediction model intended to discriminate between breast cancer patients who might respond to treatment from those who would not respond was proposed based on metabolomic profiling [14]. Another model was proposed based on lipidomics, in which lysophosphatidylcholine and sphingomyelin levels predicted cancer occurrence [15]. Such models might be useful in personalized and precision medicine.

Although mammography has been successful in screening high-risk breast cancer patients, about 20% of cases remain undetected by this technology. It is hoped that metabolomic profiling combined with other detection technologies may improve the sensitivity and specificity of cancer detection and diagnosis. Toward this end, the role of chemokine receptor CXCR4 in breast cancer aggressiveness was demonstrated by Vermeer *et al.* [16].

### 2.3. Colorectal Cancer

Colorectal cancer is one of the most prevalent and deadly cancers worldwide. Microbiome involvement, especially bacteria, has been proposed in the development of this cancer. Johnson *et al.* used colon microbiome metabolomics information to identify metabolites that can discriminate between healthy individuals and colon cancer patients [2]. Microbiota collected from the colon was analyzed for metabolites, and upregulation of polyamine and diacetylspermine metabolites was observed. Other investigators proposed using valine as a biomarker for detecting colon cancer in patients with polyps [17]. Holst *et al.* observed branched glycan elevation in colon cancer patient samples [18] and hypothesized that the interaction of proteins with colon cancer glycans contributes to carcinogenesis. Metabolomic profiling was shown to be useful in validating transcriptomic results of colon cancer samples when transcriptomic expression was functionally evaluated by metabolomic profiling [19]. Other investigators found that a combination of NMR, chemomatrix analysis of colon cancer patient serum, and comparison with controls indicated higher levels of pyridoxine, orotidine, and taurocholic acid in colon cancer samples [20]. The major metabolomic cycles involved include bile acid biosynthesis, vitamin B6 metabolism, methane metabolism, and glutathione metabolism.

### 2.4. Gastric Cancer

Although more prevalent in Asian countries, gastric cancer incidence and prevalence are increasing in Western countries [4]. Metabolomic profiling has been proposed as an alternative screening method for gastric cancer because of the high cost of endoscopy [21]. 5-Hydroxyindoleacetic acid levels were found to be higher in gastric cancer patients compared to controls [22]. Urine metabolomics were applied in this study, and the authors suggested including this urine marker along with other gastric cancer markers to improve the sensitivity of diagnostic assays.

### 2.5. Liver Cancer

Liver cancer is one of the most lethal malignancies. In the United States, the rate of liver cancer has increased by 7% during the past two decades [23]. Early detection biomarkers are needed that can be used to screen populations. The Kernel approach, a new metabolomic analysis approach, was used with liver cancer samples and reduced complications from missing values in epidemiologic studies [24]. Luo *et al.* analyzed 854 metabolite ion pairs in liver cancer samples and proposed a multiple reaction-monitoring ion pair finder that is useful in identifying new biomarkers [25]. Another study identified long-term elevated serum bile acids as potential risk factors for liver cancer [26]. Based on metabolomic profiling, Shao *et al.* identified butyrylcarnitine and hydantoin-5-propionic acid as biomarkers for diagnosing liver cancer [27]. Coen *et al.* used metabolomics profiling to evaluate the hepatotoxicity of different treatment agents [28]. Other investigators also have reported liver cancer-specific biomarkers [1]. Zheng *et al.* analyzed more than 100 serum samples from controls and liver cancer patients and identified tryptophan, glutamine, and 2-hydroxybutyric acid as early markers for liver cancer [29].

### 2.6. Lung Cancer

Cigarette smoking is the major risk factor for lung cancer, although additional factors have been identified [30]. Chronic obstructive pulmonary disease (COPD) may increase the risk of lung cancer. Metabolomic approaches were applied to distinguishing COPD and lung cancer in serum samples collected from patients with one of these diseases (TNM stages I, II, III, and IV). Higher levels of acetate, citrate, and methanol were found in individuals with COPD compared to those with lung cancer; and N-glycosylated proteins, leucine, lysine, mannose, and choline levels were higher in those with lung cancer [30].

### 2.7. Pancreatic Cancer

Blood samples from patients with pancreatic cancer and cachexia were characterized by metabolomic approaches to identify contributing metabolites [31]. Two groups, one with and another without cachexia, were followed longitudinally. Serum levels of IL-6, tumor necrosis factor (TNF)-alpha, and leptons as well as loss of body weight were determined in both groups using GC-MS. Compared to the cachexia-free group, levels of these markers varied day to day and were higher in cachexia patients. Most patients with advanced stage pancreatic cancer develop cachexia with symptoms such as decreased dietary intake, anxiety, and depression.

### 2.8. Prostate Cancer

The incidence and prevalence of prostate cancer is very high in the United States and worldwide. Although prostate-specific antigen (PSA) is used for the diagnosis and prognosis of this cancer, the sensitivity and specificity of this antigen is low. It is difficult to make a clinical decision for treatment if PSA levels are lower than 0.4 microgram per mL. Metabolomic biomarkers with potential for use in diagnosing prostate cancer include sarcosine, proline, kynurenine, uracil, and glycerol-3-phosphate in urine [32]. These metabolites can be measured in longitudinally collected urine samples by LC-MS [33].

## 3. Challenges and Opportunities

Metabolomics currently involves a variety of challenges. For example, metabolomics data generally are complex and it has been observed that the data matrix frequently contains missing values, making quantitative analysis difficult. Peaks that are present in the chromatogram can be missed by investigators during peak picking. Zhan *et al.* proposed using the Kernel-based scoring approach to address missing data [24]. The Kernel method is available in the R statistical computing environment.

Metabolomics requires the development of sophisticated and powerful statistical methodologies to make clinical observations easy to follow. These statistical approaches enable comparison of the abundance levels of a metabolite between cases and controls to assess their significance. Metabolomic association networks that use high-dimensional MS data have the potential to improve data analysis and use by investigators.

Although the analytical platforms for metabolomic analysis are robust, sample pretreatment procedures differ among institutions such as clinics and hospitals. This contributes to systematically biased results. For example, when tumor tissues are collected, normal cells may be included in these samples and confound the analysis. Laser microdissection is a potential solution, but the procedure is highly technical, time-consuming, and expensive for epidemiologic studies. Fasting status shows different metabolomic patterns in serum *versus* urine collected from the same patient. Techniques that show minimum variability of metabolites in samples from patients under fasting or non-fasting conditions are needed. Intra-individual variations have been reported for nutritional status (food frequency) and physical exercise [34].

In epidemiologic studies that seek to identify race- and ethnicity-specific metabolites, cohorts containing multiple ethnic groups are needed so that findings can be generalized to all populations. Additional challenges include: variability in metabolomic measurements and related implications; the need to create new techniques for analysis, computation, and interpretation; epidemiologists have limited training in metabolomics; the need to integrate metabolomic data with genomic, epigenomic, transcriptomic, and proteomic data; and the availability of highly purified standards.

Opportunities include the fact that biofluids such as urine and blood can be collected noninvasively and are suitable for both epidemiologic and clinical studies. Applications of metabolomics in drug-resistant breast cancer cells were described recently [35], and similar approaches can be investigated in other cancers.

## 4. Conclusions

Understanding the metabolic basis of cancer has the potential to provide the foundation for the development of novel approaches targeting tumor metabolism. Tumors characterized by aerobic glycolysis and/or glucose dependence could be more sensitive than other tumors to agents targeting the tumor vasculature and glucose transport. Tumors characterized by impaired TCA cycle function and/or respiration that is glutamine-dependent could be sensitive to agents targeting glutamine metabolism (such as glutaminase). Tumors that have impaired mitochondrial/electron transport function could be sensitive to agents that target the reductive carboxylation and fatty acid synthesis pathways. Malignancies that are characterized by IDH1, IDH2, FH, or succinate dehydrogenase mutations could affect TET2 function, resulting in hypermethylation phenotypes [36]. Such malignancies could be responsive to hypomethylating agents.

Metabolites are the end products of biological regulatory and metabolomic processes, and they can be measured in biological fluids and tissues. Their levels can be regarded as the response of biological systems to genetic, lifestyle, and environmental changes. Molecular profiling based on metabolomic analysis may facilitate the stratification of patients with cancer into homogeneous biological groups to facilitate the clinical management of these patients. Similar to other omics biomarkers, metabolomic biomarkers should have high sensitivity and specificity. Because metabolites are closely related to a patient’s phenotype, their potential for use in disease diagnosis and prognosis is significant. Metabolomics has the potential to become a valuable tool for precision medicine.
